# Mitochondrial Impairments in Peripheral Blood Mononuclear Cells of Multiple Sclerosis Patients

**DOI:** 10.3390/biology11111633

**Published:** 2022-11-08

**Authors:** María Inmaculada Domínguez-Mozo, María Celeste García-Frontini Nieto, María Isabel Gómez-Calcerrada, Silvia Pérez-Pérez, María Ángel García-Martínez, Luisa María Villar, Noelia Villarrubia, Lucienne Costa-Frossard, Rafael Arroyo, Roberto Alvarez-Lafuente

**Affiliations:** 1 Environmental Factors in Degenerative Diseases Research Group, Hospital Clínico San Carlos, Instituto de Investigación Sanitaria del Hospital Clínico San Carlos (IdISSC), 28040 Madrid, Spain; 2 Department of Immunology, Hospital Universitario Ramón y Cajal, Instituto Ramón y Cajal de Investigación Sanitaria (IRYCIS), 28040 Madrid, Spain; 3 Department of Neurology, Hospital Universitario Ramón y Cajal, IRYCIS, 28034 Madrid, Spain; 4 Department of Neurology, Hospital Universitario Quironsalud Madrid, 28223 Madrid, Spain

**Keywords:** mitochondria, multiple sclerosis, lipid-specific oligoclonal immunoglobulin M bands (LS-OCMB)

## Abstract

**Simple Summary:**

Multiple sclerosis (MS) is a chronic, inflammatory disease that affects the central nervous system, and is the most common cause of non-traumatic neurological disability in young adults. Although its origin is unknown, it is thought that the immune system damages the insulating covers of nerve cells. On the other hand, it seems that the mitochondria (the organelle producing energy in the cells) function abnormally in these patients. However, few studies focus on the mitochondria of immune cells. In this study, we compared the mitochondrial function of peripheral blood cells (PBC) from healthy donors to those of MS patients, classifying these patients by the presence (M+) or absence (M−) of lipid-specific oligoclonal immunoglobulin M bands (LS-OCMB). The detection of LS-OCMB has been associated with a highly inflammatory and more aggressive course of MS. We found signs of mitochondrial impairment in the PBCs from M+ patients compared with M− ones, which could affect in greater terms the oldest M+ patients. Currently, there is no treatment for this heterogeneous disease. The results of this study could help to understand its physiopathology better and encourage the consideration of the LS-OCMB presence in further studies delving into the effect of certain drugs.

**Abstract:**

Although impaired mitochondrial function has been proposed as a hallmark of multiple sclerosis (MS) disease, few studies focus on the mitochondria of immune cells. We aimed to compare the mitochondrial function of the peripheral blood mononuclear cells (PBMCs) from MS patients with (M+) and without (M−) lipid-specific oligoclonal immunoglobulin M bands (LS-OCMB), and healthydonors (HD). We conducted an exploratory cross-sectional study with 19 untreated MS patients (M+ = 9 and M− = 10) and 17 HDs. Mitochondrial superoxide anion production and mitochondrial mass in PBMCs were assessed without and with phytohemagglutinin by flow cytometry. The PBMCs’ mitochondrial function was analyzed using Seahorse technology. Superoxide anion production corrected by the mitochondrial mass was higher in MS patients compared with HDs (*p* = 0.011). Mitochondrial function from M+ patients showed some impairments compared with M− patients. Without stimulus, we observed higher proton leak (*p* = 0.041) but lower coupling efficiency (*p* = 0.041) in M+ patients; and under stimulation, lower metabolic potential ECAR (*p* = 0.011), and lower stressed OCR/ECAR in the same patients. Exclusively among M+ patients, we described a higher mitochondrial dysfunction in the oldest ones. The mitochondrial impairments found in the PBMCs from MS patients, specifically in M+ patients, could help to better understand the disease’s physiopathology.

## 1. Introduction

Multiple sclerosis (MS) is a chronic, demyelinating, inflammatory disease that affects the central nervous system. Although the etiology of MS is unknown, it is commonly accepted that an infectious agent could trigger the autoimmune reaction in genetically predisposed subjects. Two main core MS phenotypes have been defined: relapsing-remitting (RR) and progressive disease. Approximately 85% of people with MS are diagnosed with RRMS, characterized by clearly defined attacks of new or increasing neurologic symptoms. Among progressive diseases, we distinguished between primary progressive (PP) and secondary progressive (SP), depending on if the progressive accumulation of disability was from the onset or after the initial relapsing course, respectively [1].

Impaired mitochondrial function has been proposed as a key pathological hallmark of MS. Different studies [2] have reported mitochondrial dysfunction in these patients’ cortex and white matter, together with defects in the mitochondrial respiratory chain in their neuronal axons, astrocytes, and oligodendrocytes. Besides, this pathology has been associated with a decrease in the efficiency of oxidative phosphorylation, as well as with an increase in reactive oxygen species (ROS) production [3]. Although most of these studies are conducted at the central nervous system (CNS) level, some of them start to assess circulating immune cells. They describe the peripheral blood mononuclear cells (PBMCs) immunometabolic profile of MS patients associated with the progressive form of the disease [4], the administration or not of Interferon (IFN)-beta-1a [5], the presence of particular MS-risk polymorphism [6] or focusing on the redox status of the cells [7]. Eventually, although mitochondrial dysfunction is not only a central event in many pathologies, it contributes to age-related processes. Mitochondria have been shown to participate in every aspect of aging, such as the development of a low-grade inflammatory state, a decline in stem cell function, and cellular senescence [8].

Treatments available for MS have increased these last few years, however, diagnostic and prognostic biomarkers have not experienced the same trend. The presence of IgG in MS patients’ cerebrospinal fluid (CSF) contributes to the disease’s diagnosis. They are found in more than 95% of these patients, but they are not exclusive to this disease [9]. In contrast, the detection of intrathecal synthesis of IgM is lower (28–55%). It has been associated with a highly inflammatory and more aggressive course of MS in terms of disease activity and progression, especially the lipid-specific oligoclonal immunoglobulin M bands (LS-OCMB). Patients without LS-OCMB (M−) are twice as frequent as the patients with these bands (M+) [10,11,12,13,14,15].

Since M+ patients are associated with a worse prognosis and a more inflammatory profile, we aimed to study whether their PBMCs have mitochondrial impairments compared to healthy donors (HDs) and M− patients. This unique approach could contribute to a better understanding of this disease’s physiopathology and treat patients more personalized way.

## 2. Materials and Methods

### 2.1. Patients and Samples

We conducted a cross-sectional, observational exploratory study. We included 19 RRMS patients according to the 2017 revised McDonald criteria [9] from Hospital Universitario Ramón y Cajal (Madrid, Spain), and 17 sex and age-matched healthy donors from Hospital Clínico San Carlos (Madrid, Spain).

MS patients were not on disease-modifying treatments at the moment of the sample withdrawal. We detected the presence of LS-OCMB in approximately half of them (*n* = 9) but not in the other half (*n* = 10). The demographical and clinical characteristics of the participants at the time of sampling are shown in Table 1.

Paired serum and CSF samples obtained and stored at −80 °C were available for each enrolled patient. The serum was isolated by centrifugation. In addition, we collected a blood sample from every patient in a cell preparation tube (CPT, BD vacutainer) for PBMC isolation by centrifugation. PBMCs were cryopreserved and eventually stored in liquid nitrogen (−176 °C) until use.

### 2.2. LS-OCMB Detection

Serum and CSF IgM were quantified using an Image nephelometer (Beckman Coulter, Miami, FL, USA). LS-OCMB were studied in paired serum and CSF by isoelectric focusing and immunoblotting [16]. A patient was considered to have LS-OCMB when two or more IgM bands were detected in the CSF but not in the paired serum sample.

### 2.3. Metabolic Seahorse Assays

Once PBMCs were thawed and rested overnight, they were cultured in the presence or absence of PHA (5 ug/mL) for 24 h at 37 °C, 5% CO_2_. PHA binds to specific cell surface carbohydrates on T-cells. It can activate small lymphocytes to transform into lymphoblasts, then divide and proliferate, release cytokines, and improve the phagocytic ability of macrophages [17]. Both stimulated and non-stimulated PBMCs of every patient were washed and re-suspended in supplemented XF DMEM media supplemented with glutamine (2 mM), glucose (10 mM), plus pyruvate (1 mM), and subsequently plated (200,000 cells/well) in XFp plates for 45 min in a non-CO_2_ incubator. Stained trypan blue cells were counted using a hemocytometer. Each condition was analyzed in triplicate in a Seahorse Xfp extracellular flux analyzer (Agilent, Santa Clara, CA, USA) using Cell Mito Stress Test Kit, according to the manufacturer’s procedures. Briefly, real-time measurements of oxygen consumption rate (OCR) and extracellular acidification rate (ECAR) were obtained in basal conditions, and after oligomycin (1.5 μM; it blocks the ATP synthase), FCCP (0.5 μM; it is an uncoupler of the electron transport chain), and a cocktail of rotenone and antimycin A injections (both at 0.5 μM; they completely inhibit mitochondrial electron transport).

Data were analyzed using the Seahorse XF Cell Mito Stress Test Report Generator (selected parameters calculated from OCR profile: non-mitochondrial oxygen consumption, basal respiration, maximal respiration, proton leak, ATP-linked respiration, spare respiratory capacity (%), coupling efficiency) and the XF Cell Energy Phenotype test report generator (selected parameters: baseline and stressed OCR, baseline and stressed ECAR, metabolic potential OCR, and metabolic potential ECAR).

The forehead parameters are calculated as follows. The non-mitochondrial oxygen consumption: minimum rate measurement after rotenone/antimycin A injection; basal and maximal respiration: the difference between the last rate measurement before first injection or the maximum rate measurement after second injection respectively and the non-mitochondrial oxygen consumption; proton leak: subtraction of the non-mitochondrial oxygen consumption to the minimum rate measurement after first injection; ATP production: difference between the last rate measurement before and minimum rate mea-surement after first injection; spare respiratory capacity: maximal minus basal respiration; coupling efficiency: ATP production rate divided by the basal respiration rate; baseline OCR and ECAR: last measure before oligomycin injection; stressed OCR: maximal OCR in all experiment; stressed ECAR: maximal ECAR between after and before oligomycin and FCPP injection respectively; metabolic potential: percentage increase of stressed OCR over baseline OCR, and stressed ECAR over baseline ECAR.

### 2.4. Flow Cytometry

For mitochondrial superoxide anion measurement, PBMCs (stimulated or not with phytohemagglutinin (PHA)) were incubated with 5 µM MitoSOX red (Thermo Fisher, Waltham, MA, USA) for 20 min at 37 °C, 5% CO_2_.

For assessment of mitochondrial mass, PBMCs (stimulated or not with PHA) were surface stained with the following antibodies: anti-CD8-PC7, anti-CD19-APC, anti-CD4-APC, anti-CD3-APC-Cy7, and 7AAD for 15 min at room temperature in the dark. Then, the labeled PBMCs were washed and incubated with 0.1 µM MitoTrackerGreen (MTG) (Thermo Fisher, Waltham, MA, USA) for 30 min at 37 °C, 5% CO_2_. This mitochondria-specific dye binds the mitochondrial membrane independently of membrane potential and is considered an index of mitochondrial mass [18].

A gate including lymphocytes and excluding debris was established. Next, we exclude non-viable cells. Cells were analyzed in a CytoFLEX flow cytometer (Beckman Coulter, Miami, FL, USA), and data were parsed with CytExpert Software.

### 2.5. Statistical Analysis

Numerical variables were expressed as mean ± SD (standard deviation) or median (25th, 75th percentile), and categorical variables as percentages. Categorical variables were compared using the chi-squared or Fisher’s exact test, and numerical variables between groups using the Mann–Whitney U test. *p*-values < 0.05 were referred to as statistically significant in the text. All analyses were performed using SPSS version 21.0 (IBM), and graphs were made using Prism version 5.0 (GraphPad Prism, San Diego, CA, USA).

## 3. Results

### 3.1. Mitochondrial Mass from CD4+ T Cells, CD8+ T Cells, and B Cells under Stimulus Was Higher than without Stimulus

Regarding the percentage of lymphocytes subpopulation, we found that the percentage of B lymphocytes was higher in MS patients than in HDs, in both conditions, under PHA stimulation and no stimulation. Exclusively under PHA stimulation, the CD4+ T lymphocyte percentage was higher in HDs vs. MS patients. Finally, under no stimulation, the percentage of CD8+ T lymphocytes was higher in M+ than in M− (Figure S1).

**Figure 1:**
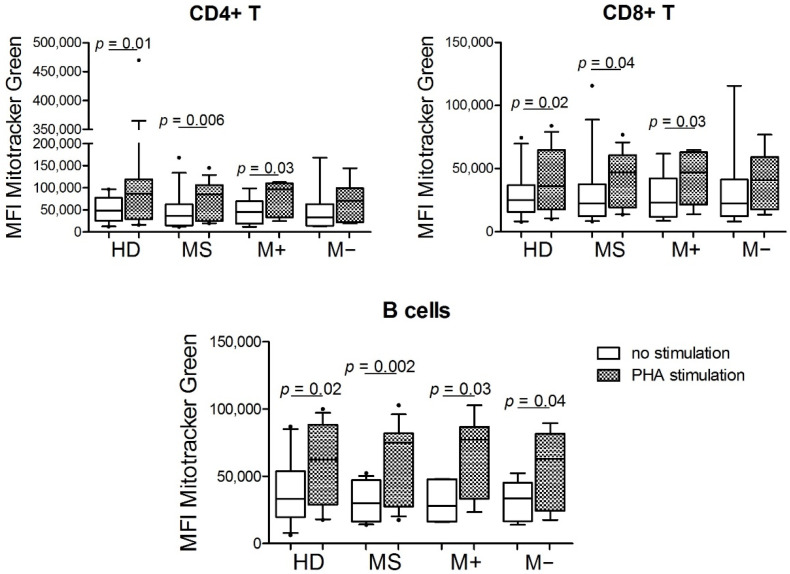
Mitochondrial mass of cell subsets from peripheral blood of multiple sclerosis (MS) patients and healthy donors (HD).

We assessed the mitochondrial mass of CD4+ T cells, CD8+ T cells, and B cells from 16 RRMS patients (7 with and 9 without LS-OCMB) and 13 HDs measuring the mean fluo-rescence intensity (MFI) of MTG dye. We did not find any difference between the MFI of the HDs and the MS patients, nor between the M+ vs. M− patients. Since PHA is used as a T lymphocyte proliferation inducer, we observed higher mitochondrial mass in CD4+ T cells, CD8+ T cells, and B lymphocytes under PHA stimulation compared to those under no stimulus in overall MS patients, M+, and HD. However, among the M− patients, we only observed this increase in their B cells (Figure 1). MFI of MTG was not associated with the age of the participants.

The mitochondrial mass of CD4+ T, CD8+ T, and B cell subsets was assessed by staining with Mitotracker Green dye, under PHA stimulation (box with pattern) vs. no stimulus condition (box without pattern). MFI: Mean Fluorescent Intensity. M+: MS patients with lipid-specific oligoclonal immunoglobulin M bands (LS-OCMB) in cerebrospinal fluid; M−: MS patients without LS-OCMB. P-values were calculated using the Wilcoxon t-test. HD = 12, MS = 14, M+ = 6, M− = 8. PHA = phytohemagglutinin. In the box plots, the box boundary closest to zero indicates the 25th percentile, the line within the box marks the median, and the box boundary farthest from zero indicates the 75th percentile. Whiskers above and below the box indicate the 10th and 90th percentiles.

### 3.2. PBMCs from MS Patients Had Greater Superoxide Production than Those from HDs

**Figure 2:**
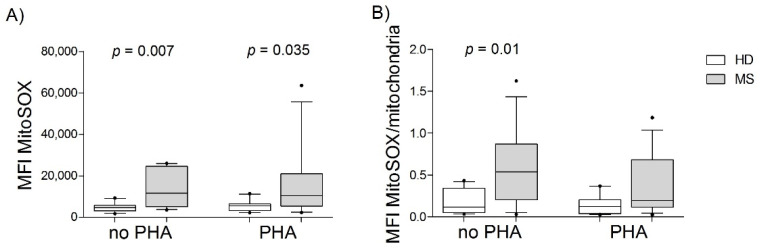
Mitochondrial ROS in PBMCs from multiple sclerosis (MS) patients and healthy donors (HD). (**A**) Mitochondrial superoxide anion production was measured using MitoSOX by flow cytometry. (**B**) Mitochondrial superoxide anion production was analyzed per mitochondrial content (MitoSOX/Mitotracker Green). No PHA: HD = 12, MS = 12; PHA: HD = 12, MS = 14. MFI: mean fluorescent intensity; PHA = phytohemagglutinin. P-values were calculated using the Mann-Whitney U test. In the box plots, the box boundary closest to zero indicates the 25th percentile, the line within the box marks the median, and the box boundary farthest from zero indicates the 75th percentile. Whiskers above and below the box indicate the 10th and 90th percentiles.

When possible, we measured the superoxide anion (the predominant ROS in mitochondria) using the MitoSOX Red reagent in the PBMCs from the participants previously mentioned (13 HD, 6 M+, and 9 M−). The MFI of this dye was higher in the mitochondria of MS patients than in those of HDs under no stimulation (*p* = 0.007) and PHA stimulation (*p* = 0.035) (Figure 2A). When we corrected the superoxide anion values by the mitochondrial mass (MitoSOX/Mitotracker Green), we only found differences in non-stimulated cells (*p* = 0.011) (Figure 2B). We did not find any association between the age of the parti cipants and the levels of superoxide production.

### 3.3. Signs of Functional Mitochondrial Impairments among M+ Patients

**Figure 3:**
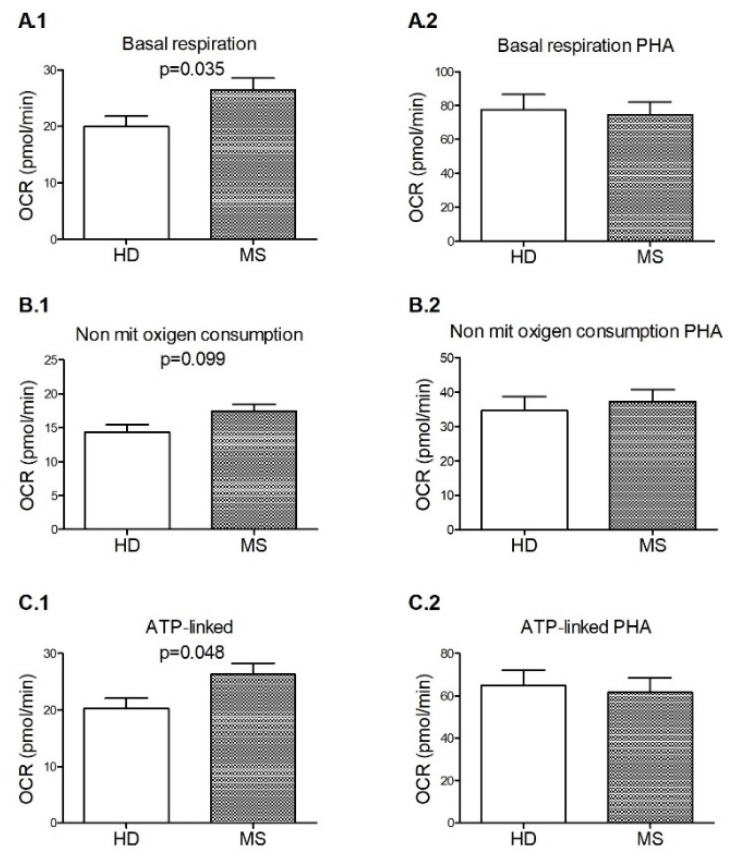
Impaired mitochondrial respiration in the PBMCs (peripheral blood mononuclear cells) from healthy donors (HD) vs. multiple sclerosis (MS) patients. Indexes of mitochondrial respiratory function were calculated from the OCR profile of non-stimulated (**1**) and PHA-stimulated (**2**) PBMCs: basal respiration (**A**), non-mitochondrial oxygen consumption (**B**), and ATP-linked (**C**). Healthy donors (HD) *n* = 17; Multiple sclerosis patients (MS) *n* = 18. OCR: oxygen consumption rate; PHA = phytohemagglutinin. Data were expressed as mean ± s.e.m. Comparisons were evaluated with the non-parametric test U Mann-Whitney.

We assessed the glycolysis and mitochondrial respiration by analyzing the extracellular ECAR and OCR of PBMCs isolated from HD and untreated MS patients under non-stimulus and PHA stimulation conditions (Figure 3). In non-stimulated PBMCs, we observed that the basal respiration (*p* = 0.035) and the ATP production (*p* = 0.048), both indicators of the energetic demand of the PBMCs under baseline conditions, were higher in RRMS patients compared with HD. In addition, we observed the same trend regarding non-mitochondrial oxygen consumption (*p* = 0.099). However, these differences were not observed under PHA stimulation. No differences were observed regarding maximal respiration, proton leak, spare respiratory capacity, coupling efficiency, baseline and stressed OCR, baseline and stressed ECAR, metabolic potential OCR, or metabolic potential ECAR between HD and MS patients.

**Figure 4:**
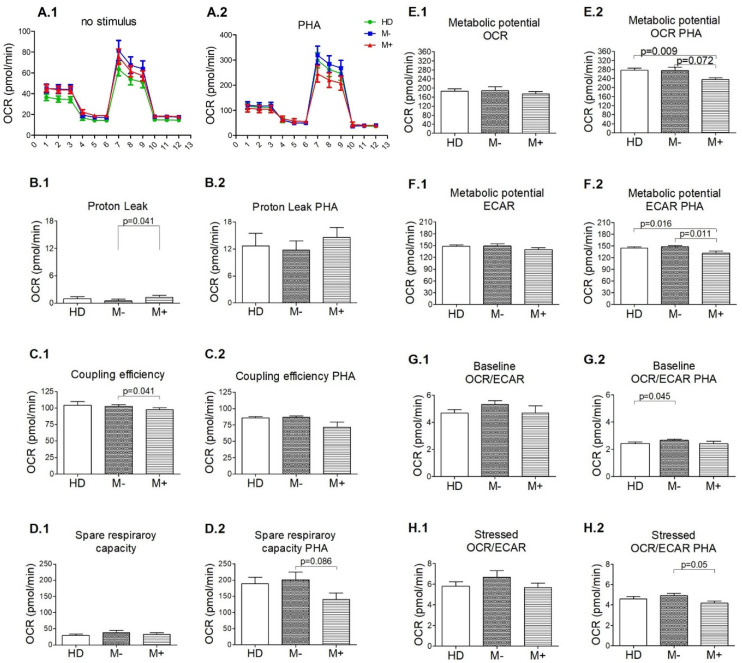
Mitochondrial dysfunction in the peripheral blood mononuclear cells (PBMCs) from multiple sclerosis (MS) patients with lipid-specific oligoclonal immunoglobulin M bands (LS-OCMB) (M+). (**A**) Kinetic profile of oxygen consumption rate (OCR) of non-stimulated (**1**) and PHA-stimulated (**2**) PBMCs was measured in real-time under basal conditions and in response to mitochondria inhibitors: oligomycin, FCCP, and Antimycin A plus Rotetone. (**B**,**C**) The indexes of mitochondrial function were calculated from the OCR and extracellular acidification rate (ECAR) profiles of non-stimulated (**1**) and PHA-stimulated (**2**) PBMCs: proton leak (**B**), coupling efficiency (**C**), spare respiratory capacity (**D**), metabolic potential OCR (**E**), metabolic potential ECAR (**F**), baseline OCR/ECAR (**G**) and stressed OCR/ECAR (**H**). Healthy donors (HD) *n* = 17; M+: MS patients with lipid-specific oligoclonal immunoglobulin M bands (LS-OCMB) in cerebrospinal fluid *n* = 10; M−: MS patients without LS-OCMB *n* = 9; PHA = phytohemagglutinin. Data were expressed as mean ± s.e.m. Comparisons were evaluated with the non-parametric test U Mann-Whitney.

In addition, we assessed the mitochondrial function of M+ patients compared with M− (Figure 4). Under no stimulus, the PBMCs from M+ patients had a higher proton leak (a sign of mitochondrial damage) (*p* = 0.041) but a lower coupling efficiency (*p* = 0.041) than M− patients. Similarly, after PHA stimulation, the metabolic potential ECAR, an indicator of cells’ ability to meet an energy demand via glycolysis, was lower among M+ patients vs. M− patients (*p* = 0.011) and vs. HD (*p* = 0.016). Also, under stimulation, Spare respiratory capacity (a measure of the ability of the cell to respond to increased energy demand or under stress) (*p* = 0.086), metabolic potential OCR (*p* = 0.072), and the stressed OCR/ECAR (*p* = 0.05) showed a trend to be lower in the M+ patients compared with M−. Moreover, metabolic potential OCR was statistically lower (*p* = 0.009) among M+ patients than among HD. Also, the baseline OCR/ECAR was exclusively lower in HDs vs. M− patients (*p* = 0.045). On the other hand, most of the parameters reported by the Seahorse XF Cell Mito Stress Test increased after PHA stimulation compared with no stimulation. However, the significance level among M− patients was higher (*p* = 0.005) than among M+ patients (*p* = 0.012) but not higher than among HD (*p* = 0.0002).

**Figure 5:**
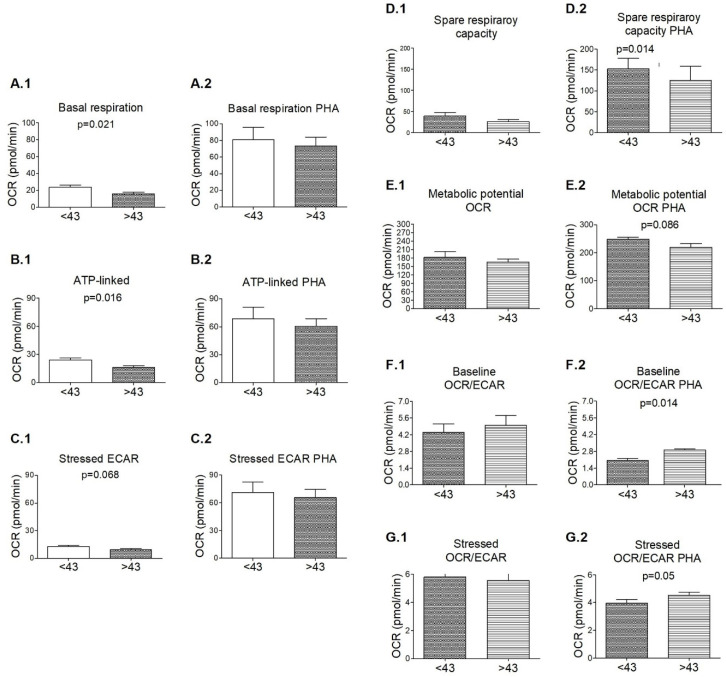
Mitochondrial function of the PBMCs from (**A**) healthy donors (HDs) and (**B**) MS patients with lipid-specific oligoclonal immunoglobulin M bands (LS-OCMB) (M+) depending on age. The indexes of mitochondrial function were calculated from the oxygen consumption rate (OCR) and extracellular acidification rate (ECAR) profiles of non-stimulated (**1**) and PHA-stimulated (**2**) PBMCs: Basal respiration (**A**), ATP-linked (**B**), stressed ECAR (**C**), spare respiratory capacity (**D**), metabolic potential OCR (**E**), baseline OCR/ECAR (**F**) and stressed OCR/ECAR (**G**). Healthy donors (HDs) younger than 43 years old (<43) = 9, HDs older than 43 years old (>43) = 8. M+ < 43 = 5, M− > 43 = 4. PHA = phytohemagglutinin. Data were expressed as mean ± s.e.m. Comparisons were eva-luated with the non-parametric test U Mann-Whitney.

Finally, we analyzed if age could affect the mitochondrial functionality of both HD and MS patients. Because of the small number of individuals, we consider the mean age of all our population (42.9 ± 10.5 years old) as the reference value to distinguish between young and aged participants. Among HDs, basal respiration (*p* = 0.021) and ATP production (*p* = 0.016) were statistically higher in the younger population; we found the same trend (*p* = 0.068) for the stressed ECAR, always in non-stimulated PBMCs (Figure 5A). While we did not find any difference between the bioenergetic state of the mitochondria of younger vs. aged M− patients, we found it among the PBMCs under PHA stimulation from M+ patients. The spare respiratory capacity (*p* = 0.014), and the baseline OCR/ECAR (*p* = 0.014) were higher and lower respectively among the patients younger than 43 years old. We found a trend to higher and lower values of metabolic potential baseline OCR (*p* = 0.086) and the stressed OCR/ECAR (*p* = 0.05) of younger participants vs. the aged ones (Figure 5B). Moreover, we observed a strong negative correlation (*n* = 9) of age with me- tabolic potential OCR (r = −0.778, *p* = 0.014), spare respiratory capacity (r = −0.82, *p* = 0.07), and a positive one with baseline OCR/ECAR (r = 0.879, p = 0.002) and stressed OCR/ECAR (r = 0.69, *p* = 0.058). Whereas in M− patients, we did not observe any significant correlation; among HDs, a moderate negative correlation between age and basal respiration (r = −0.599, *p* = 0.01); and ATP production (r = −0.553, *p* = 0.021).

## 4. Discussion

The recent scientific literature describes mitochondrial dysfunction in the PBMCs from patients with autoimmune diseases [19]. However, only a few studies have been published on MS, and none considers the degree of inflammation associated with this pathology reflected by the absence or presence of LS-OCMB in the CSF of these patients. This exploratory study shows mitochondrial impairment in the PBMCs of M+ patients compared with M− patients, together with higher levels of ROS anion in patients vs. HD. This could be helpful to better understand the pathophysiology of this disease.

We did not find statistical differences between the mitochondrial mass of HD and MS patients, or between M+ and M− patients. These results are difficult to compare with pre- vious ones; Biassi et al. [4] assessed the mitochondrial mass in CD4+ T cells from progressive MS patients. After in vitro stimulation, the authors found a lower mitochondrial mass in CD4+ T cells from PP patients compared with SP ones; they did not find any difference with the control group and did not include RR patients. We observed that mitochondrial mass increased upon PHA stimulation in HD and MS patients. We detected this augment in B cells from M+ and M− patients. However, we only detected this increase in CD4+ T and CD8+ T cells from M+ patients. Given that M+ patients have a more inflammatory profile, a higher increase of mitochondria could be necessary to cover a higher energy demand compared with M− patients. For instance, an easier activation and differentiation of naïve T cells to the same stimulus would be determined by mitochondrial mass [4].

The MS group showed a significant increase in superoxide production compared with HDs. This increase in cellular superoxide production has also been described in cardiovascular diseases [20] and neurodegenerative diseases such as amyotrophic lateral sclerosis [21], Alzheimer’s [22], and Parkinson’s diseases [23]. The superoxide anion, a very reactive ROS, reacts with DNA, lipids, and proteins, causing harmful effects on cells and interacting with other elements to spread new ROS. Mitochondrial superoxide anion has been shown to activate T cells and is considered a critical second messenger for TCR signaling [24]. In addition, it seems to be key for synthesizing IL-2 and expressing specific surface markers, such as CD69 and CD25 [24]. The detected ROS increase could reflect the higher activation level of T cells typical of MS patients even without stimulus. A previous study demonstrated higher levels of CD69 in T cells from MS patients [6], which could be mediated, at least in part, by the increase of mitochondrial superoxide anion after PHA stimulation. Although this increase was observed in non-stimulated and PHA-stimulated cells, after being corrected by mitochondrial mass, only in the non-stimulated condition remained significant. Under PHA stimulation, this increase in ROS production would be counteracted by an augmentation of mitochondrial mass, which would quench the damaging effects of the superoxide anion. Gonzalo et al. [7] found this higher ROS production among non-stimulated PBMCs from RRMS patients (76.5% of them with disease-modifying therapies (DMTs)) compared with controls. In contrast, Biasi et al. [4] found that the ROS production in subpopulations of T cells from both controls and progressive MS patients remained low before and after 30’ of in vitro stimulation. None of these two studies measured mitochondrial mass.

Previous studies [25] showed that an impaired state of the mitochondria is associated with lower basal respiration, mainly caused by an increase in non-mitochondrial respiration and a decrease in ATP-linked respiration. We found higher basal respiration among our patients, contrary to what would be expected. However, it is caused by a higher ATP-linked level, probably related to the higher demand for energy associated with the inflammatory status of this autoimmune disease. The PBMCs of our MS patients likely have a sub-healthy mitochondrial population, still capable of meeting the cell’s energy demand, although they start to accumulate some functional defects, such as the trend to have higher non-mitochondrial respiration found among the MS patients. These last data are in agreement with the higher mitochondrial superoxide production found among our MS patients. In fact, under PHA stimulation basal respiration and ATP-link are no longer higher among MS patients; pointed out that PBMCs of both cohorts would react similarly to fa-cing an extra demand of energy and the MS mitochondrial function is not better. However, previous studies [5] described a decreased basal, maximal respiration, and spare capacity in stimulated PBMCs from naïve-to-treatment MS patients compared to HD. The discrepancy between these results could be due to the different stimuli, PHA in our study and anti-CD3 in the previous one, and, perhaps more importantly, they did not explore patient LS-OCMB status, associated with a high inflammatory and more aggressive course of MS [12,13,14,15].

Grouping our patients depending on the presence of LS-OCMB, we observed more signs of impairment in the mitochondria of the PBMCs from M+ patients, such as a higher proton leak but lower coupling efficiency compared with M− patients. These results agree with the higher inflammatory profile associated with M+ patients [16], which could lead to a certain degree of lymphocyte exhaustion even in a baseline status. Chronic metabolic stress induce damage in the mitochondrial respiratory machinery, progressively decreasing mitochondrial function. This could explain a higher response (assessed with the p-value) to PHA among M− patients compared with M+ ones. Additionally, under in vitro stimulation with PHA, we found a lower ability to meet an energy demand via glycolysis or via respiration in M+ patients compared with M− ones and HD. However, in M+ patients, we observed a higher capability to upregulate their metabolic activity increasing ECAR when their PBMCs are in the presence of stressor compounds compared with M− ones.

Well-documented evidence suggests a close relationship between the systemic me-tabolic asset and immune function [26]. Once activated, the equilibrium between mitochondrial and glycolytic energy production controls T-cell fate [27]. Whereas Tregs primarily rely on lipid oxidation, effector T cells preferentially utilize glycolysis as a means of energy generation [26]. We could speculate that the low stressed OCR/ECAR ratio observed in the PBMCs from M+ patients could be associated with higher activation of the effector T cell compartment via glycolysis. Future studies recruiting more patients are warranted to determine how the OCR/ECAR ratio varies in every T cell subpopulation depending on the presence or absence of LS-OCMB in RRMS patients. This could also explain the discrepancies described in previous studies about the increased [28] or decreased [5] glycolysis in T cells from MS patients.

Accumulating evidence suggests that age increases mitochondrial dysfunction. In addition, aging seems to be a risk factor for MS progression. It reduces the capability of the CNS to remyelinate and increases chronic microglial activation [29]. We considered age as a relevant factor to evaluate. The effect that aging has on the immune system has been named “immunosenescence”. It is characterized by chronic activation of the innate immune response and lower effectiveness of the adaptive response. Although this phenomenon occurs in people older than 65, it arises earlier in patients with chronic immune-system activation, such as rheumatoid arthritis and acquired immunodeficiency syndrome [30]. This could agree with our findings in HDs; we did not observe signs of mitochondrial impairment since we considered an age more or equal to 43 years to identify older individuals. However, as expected, basal respiration was higher in younger than in older individuals. This could be associated with the greater ATP-linked in younger po-pulations; both parameters seemed to decrease with age. Additionally, the younger po-pulation showed a trend to have a higher level of glycolysis, which is necessary for human T-cell activation under stress [31]. Whereas in M− patients, age does not seem to affect the mitochondrial functionality, M+ patients younger than 43 years showed greater spare respiratory capacity than the older ones, and a higher capacity to switch from a metabolism mainly based upon mitochondrial respiration to a metabolism where the glycolytic flux is prevalent, what is necessary to cell activation upon a stimulus such as PHA [32]. In view of these results, we hypothesize that PBMCs of M+ patients could have signs of exhaustion, as a consequence of the higher inflammation that characterizes them. Although previous studies point out that premature immunosenescence affects patients with auto- immune diseases, most do not propose a reference age. However, a recent publication [33] study this phenomenon classifying the 263 MS patients according to their age in subgroups of five years (i.e., ≤25, 26–30, 31–35 years, and so on). Eventually, these authors sorted patients into two groups: age ≤45 or > 45 years because the most significant changes in CSF lymphocyte numbers among M− patients were observed at this age, 45 years old. The authors observed that the main effect of aging in M− was a decrease in the absolute numbers of CSF T cells (including Th1 and Th17 cells) and B cells (including TNF-alpha producers) with no augment of TIM−3 levels, being the only indication of immunosenescence a discrete increase of anti-CMV antibody titers [33]. By contrast, M+ patients show an age-associated increase of TIM−3, a biomarker of T cell exhaustion [34,35]. In agreement with this, in the present study, only M+ patients older than 43 years seemed to present signs of early immunosenescence at the functional mitochondrial level.

## 5. Conclusions

In summary, we found higher ROS production among MS patients compared with HD, also signs of mitochondrial impairment in the PBMCs from M+ patients compared with M− ones, which could affect in greater terms those M+ patients aged more than 43 years; together with a tendency to upregulate glycolysis vs. mitochondrial respiration when facing a stimulus. These differences should be considered when analyzing the effect of drugs affecting metabolism, such as IFN beta-1a [5], teriflunomide [36], dimethyl fumarate [37], and BTK [38]. Understanding the bioenergetic changes in the PBMCs of MS patients could be key to identifying new biomarkers and therapeutic targets in the disease.

## Figures and Tables

**Table 1:** Demographic and clinical characteristics of the patients and healthy donors included in this study.

	Non-Treated Multiple Sclerosis Patients		Healthy Donors (*n* = 17)
	M− (*n* = 10)	M+ (*n* = 9)	
Age (years, median (P25, P75)	43 (37.8, 49.5)	39 (34.5, 46)	42 (37, 49.5)
Sex (M/F) (%)	30/70	11/89	24/76
Disease duration (years, median (P25, P75)	9.8 (1.1, 19.3)	5.8 (2.2, 15.9)	
EDSS	1.5 (1.5, 6.5)	1.5 (1, 5.8)	
Relapses 6 months before	0 (0, 1)	1 (0, 1.5)	
Relapses 6 months after	0 (0, 0)	0 (0, 0)	

EDSS: Expanded Disease Scale Status; F = female; M−: patients without lipid-specific oligoclonal immunoglobulin M bands (LS-OCMB); M+: patients with LS-OCMB; M: male. Continuous variables are expressed as median (25th, 75th percentile).

## Data Availability

The data supporting this study’s findings are available from the corresponding author upon reasonable request.

## Supplementary Materials

The following supporting information can be downloaded at: https://www.mdpi.com/article/10.3390/biology11111633/s1, Figure S1: Comparison of major lymphocytes subpopulations distribution between (A,B) healthy donors (HD) and multiple sclerosis patients (MS), and (C) MS patients without (M−) and with (M+) lipid-specific oligoclonal immunoglobulin M bands.
