# Supplementary material for: Mitochondrial Impairments in Peripheral Blood Mononuclear Cells of Multiple Sclerosis Patients
Source: Biology (Basel). 2022 Nov 8;11(11):1633. doi: 10.3390/biology11111633 (PMC9687791; doi:10.3390/biology11111633)
Supplement: Supplementary file 1 [file biology-11-01633-s001.zip › biology-1978950-supplementary.pdf]

biology

MDPI

Supplementary material

Figure S1

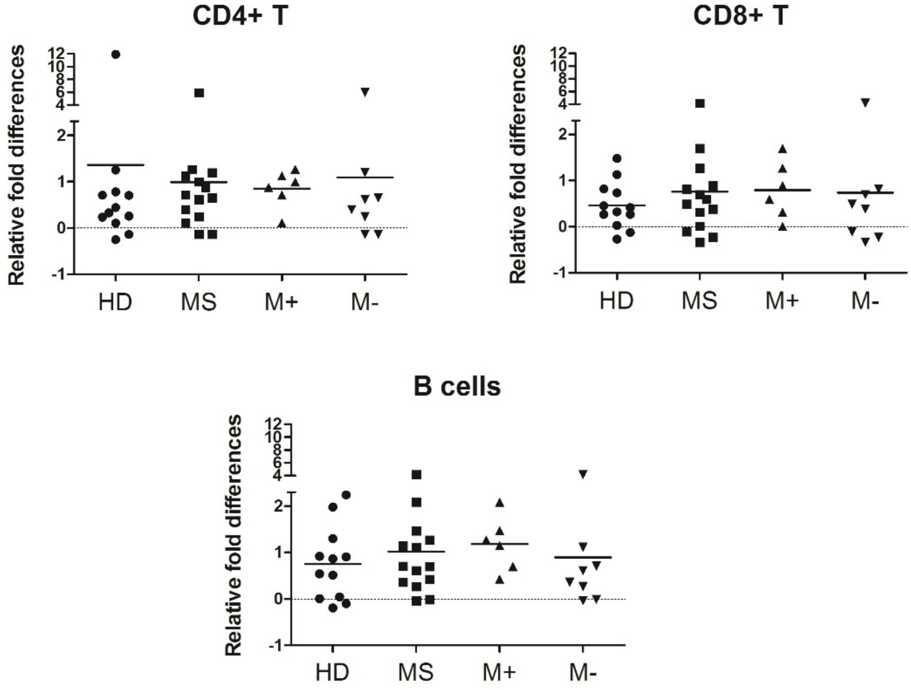

Figure S1. Comparison of major lymphocytes subpopulations distribution between (A, B) healthy donors (HD) and multiple sclerosis patients (MS), and (C) MS patients without (M-) and with (M+) lipid-specific oligoclonal immunoglobulin M bands. (A, B) HD=12, MS=15. (C) M-=9, M+=6. PHA=phytohemagglutinin. P-values were calculated using Mann-Whitney U test. In the box plots, the box boundary closest to zero indicates the 25th percentile, the line within the box marks the median, and the box boundary farthest from zero indicates the 75th percentile. Whiskers above and below the box indicate the 10th and 90th percentiles.
